# Advances in Nutrition Science and Integrative Physiology: Insights From Controlled Feeding Studies

**DOI:** 10.3389/fphys.2019.01341

**Published:** 2019-10-29

**Authors:** Kevin P. Davy, Brenda M. Davy

**Affiliations:** Department of Human Nutrition, Foods, and Exercise, Virginia Tech, Blacksburg, VA, United States

**Keywords:** controlled feeding study, dietary intake, biomarker, energy balance, weight gain

## Abstract

Nutrition science is a highly impactful but contentious area of biomedical science. Establishing cause and effect relationships between the nutrients and/or diets we consume and the avoidance of or risk of disease is extremely challenging. As such, evidence-based nutrition is best served by considering the totality of evidence across multiple study types including nutritional epidemiological studies, randomized controlled trials of behavioral interventions, and controlled feeding studies. The purpose of the present review is to provide an overview for those conducting research outside of clinical nutrition on how controlled feeding studies can be used to gain insight into integrative physiology/metabolism as well as to inform dietary guidelines. We discuss the rationale, basic elements, and complexities of conducting controlled feeding studies and provide examples of contributions of controlled feeding studies to advances in nutrition science and integrative physiology. Our goal is to provide a resource for those wishing to leverage the experimental advantage provided by controlled feeding studies in their own research programs.

## Nutritional Science Is a Highly Impactful Area of Biomedical Science

Nutrition science is a highly impactful area of biomedical science for the primary reason that the amount and composition of the diet has a profound influence on disease risk (Schwingshackl et al., 2018). In fact, eight of the 25 most-mentioned scientific papers of 2018 were focused on nutrition/dietary intake (Altmetrics, 2018). The two most read articles in the Journal of the American Medical Association Internal Medicine in 2018 were related to nutrition (Baudry et al., 2018; Loftfield et al., 2018). Dietary intake impacts disease risk through the actions of energy (i.e., calories) and nutrients on physiological function. Excesses and deficiencies of energy or a variety of nutrients can lead to physiological dysfunction, disease, and even death. Importantly, nutrition plays a crucial role throughout the life cycle from before birth and continues to affect us throughout life depending on our selected diet. Finally, despite its importance in physiological function, nutrition receives inadequate attention in many graduate physiology and medical education programs (Devries et al., 2019); greater than 60% of internal medicine residents report receiving little or no training in nutrition (Khandelwal et al., 2018). In general, nutrition is often an overlooked variable in physiology research. As such, there is considerable opportunity to improve our understanding and teaching of physiology by carefully considering dietary factors as causal agents or confounds.

The Dietary Guidelines for Americans (DGA) forms the basis for federal nutrition policy and nutrition education (U. S. Department of Health and Human Services and U. S. Department of Agriculture, 2015). The current report (DGA 2015–2020) recommends that Americans follow a healthy eating pattern that includes a variety of fruits and vegetables, fat-free or low-fat dairy, whole grains, a variety of proteins, and healthy oils. The guidelines also recommend limiting calories from added sugars and saturated fats, and reducing sodium intake. The Dietary Approaches to Stop Hypertension (DASH) diet is specifically mentioned as one healthy eating pattern that could be followed to fulfill those recommendations.

Poor diet is associated with early death and disability in the United States and around the world. Approximately 5,30,000 deaths were related to poor diet in 2016, and greater than eighty percent of these were attributable to cardiovascular diseases (CVD) (United States Burden of Disease Collaborators Mokdad et al., 2018). In addition, poor diet was the third leading risk factor for disability overall following tobacco use and high body mass index. There have been improvements in some aspects of the diet, particularly reductions in *trans* fatty acid and sugar sweetened beverage intake, and these changes have been associated with reduced disease burden and lower premature death (Wang et al., 2015). However, overall diet quality remains poor. Nevertheless, there remains considerable debate regarding the validity of the DGA and nutrition science which, in general, has become a highly contentious field (Forouhi et al., 2018; Ludwig et al., 2018a, b; Mozaffarian and Forouhi, 2018).

Establishing cause and effect relationships between the nutrients and/or diets we consume and the avoidance of or risk of disease is extremely challenging. As such, evidence-based nutrition is best served by considering the totality of evidence across multiple study types including nutritional epidemiological studies, randomized controlled trials of behavioral interventions, and controlled feeding studies (Navia et al., 2010; Magni et al., 2017; Mozaffarian and Forouhi, 2018). The purpose of the present review is to provide an overview for those conducting research outside of clinical nutrition on how controlled feeding studies can be used to gain insight into integrative physiology/metabolism as well as to inform dietary guidelines. Additional resources for further reading are also provided.

## Rationale, Basic Elements and Complexities of Controlled Feeding Studies

Dietary intake influences numerous metabolic and physiological processes. Although self-reported dietary intake assessment methods are frequently used in biomedical research, the limitations of self-reported methods are widely acknowledged (Archer et al., 2015; Davy and Estabrooks, 2015; Subar et al., 2015). Controlled feeding studies are needed to determine cause-and-effect relationships between dietary intake and physiological or health outcomes [e.g., sodium intake and blood pressure (BP)], and/or to control for the potential confounding effects of differences in dietary intake on outcomes of interest (e.g., inulin supplementation and glucose tolerance). In addition, the ability to perform deep phenotyping in this context is a significant strength of this experimental approach.

### Design Issues

The study protocol, including dietary aspects, should be designed to test a well-founded hypothesis with clearly defined outcomes. The duration or time needed to produce the expected effects, whether to use a parallel or crossover randomized controlled trial design, participant characteristics, type of diet needed (e.g., standardized diet vs. altered macro/micro-nutrient composition), and other potential confounding factors that must be accounted for (e.g., dietary supplement use, phytochemicals) are all important factors to consider when designing a controlled feeding study. Available resources must also be considered given that controlled feeding studies are time- and resource-intensive. Cost considerations include the need for research-quality software used to design controlled diets (e.g., NDS-R, ProNutra); food and paper supplies (e.g., $25–30/participant/day); trained staff to design, prepare and provide study diets daily; as well as food preparation and storage equipment and capabilities.

### Menu Development

After the controlled feeding dietary parameters have been established, such as macro/micro-nutrient targets, a research dietitian can utilize nutrition software and food composition databases to develop menus. An acceptable degree of precision (target amounts vs. menu amounts) can be determined in advance, based upon study needs (Mitchell et al., 2015). A 3- to 7-day repeating cycle of menus can be used, in order to minimize the variety of study foods needed as well as the amount of menu development required. Longer menu cycles do not appear to improve dietary compliance (Hall and Most, 2005). Menus can be planned in advance at a range of calorie levels (Mitchell et al., 2015), and adjusted as needed to meet individual energy requirements. Foods should be selected which are palatable and familiar to the target study population (e.g., children vs. older adults), and consistently available to study personnel through local vendors. Using some pre-prepared convenience items can reduce food preparation time and staffing resources, if needed. Once menus have been developed, the nutrient composition can be verified by chemical analysis (Most et al., 2003), although this requires unique laboratory capabilities. Commercial laboratories may provide a cost-effective alternative when needed infrastructure and/or expertise is lacking.

### Preparing and Providing Diets

After menus are developed and foods procured, daily food preparation forms should be developed with gram amounts and preparation instructions for each food listed. Study staff involved in food preparation should undergo safe food handling training, such as ServSafe food handler training and certification^1^. Once prepared, daily diets for each participant can be packed into portable cooler bags, picked up daily by (or delivered to) participants, and consumed off-site. Dietary provision quality assurance can be monitored by random checks of study cooler contents relative to food preparation forms, by supervisory study personnel.

**FIGURE 1:**
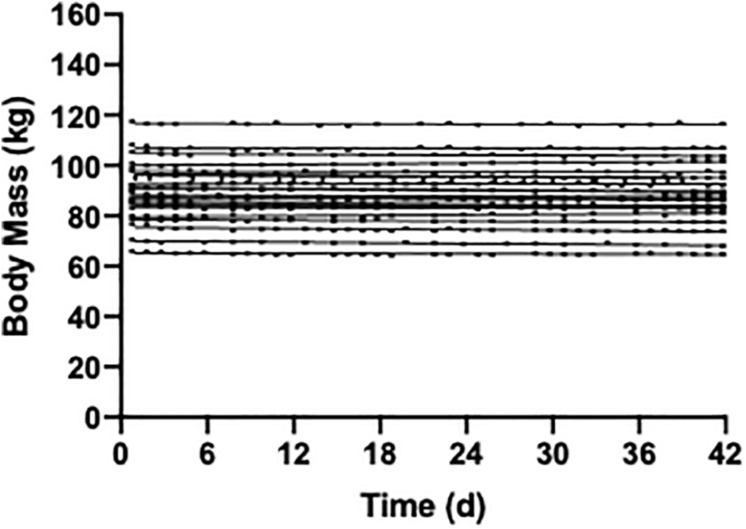
Weight stability over a 42 day period of controlled feeding in 22 participants (unpublished data). The diet composition was 55% carbohydrate, 30% fat, and 15% protein and isocaloric to each individuals energy requirements.

A variety of approaches can be used to determine participant’s total daily energy needs, including prediction equations and estimated activity level (Flack et al., 2016), indirect calorimetry combined with actigraphy, and doubly labeled water (Lam and Ravussin, 2016; Lam and Ravussin, 2017). To account for day-to-day variation in participant’s activity level and thus energy needs, optional 250–300 kcal food module “snacks” can be provided, which match the macro/micro-nutrient proportions of the controlled diet (Mitchell et al., 2015). During controlled feeding periods, participants should be weighed daily to ensure body weight stability (see Figure 1), or to monitor the desired trajectory of body weight if the protocol entails intentional weight loss or gain. Increases or decreases in body weight, if not intentional, should be addressed through modifications in the energy level of the controlled diet. Body weight loss or gain can influence many physiological outcomes, and thus confound results. As such, attention to weight stability is critical.

### Assessing Compliance

In-patient controlled feeding approaches are ideal for close monitoring of dietary compliance. If this is not feasible, methods exist to insure compliance in free-living situations are possible. Although participants are instructed to eat all provided foods, and not to consume foods outside of what is provided to them, verifying compliance is a critical issue. This may be more challenging in some age groups than others, such as young adults (Most et al., 2003). Some protocols allow one self-selected meal per week, or alcohol consumption, however, these approaches may actually worsen dietary compliance (Hall and Most, 2005). Study staff should establish a good rapport with study participants, and encourage honest self-reporting of dietary compliance. Uneaten study foods can be returned, weighed, and documented on meal preparation forms. Deviations from the study diet can also be documented, in order to calculate actual intake compared to controlled diet targets. Study protocols can also require participants to consume at least one meal daily that is supervised by research staff at the study site (Most et al., 2003; Mitchell et al., 2015).

In addition to self-reports, objective indicators of controlled diet compliance can be utilized. This may include dietary biomarkers, such as an assessment of urinary sodium or nitrogen excretion which can be compared to the amounts provided in study diets (Moore et al., 2017), or other biological markers such as para-aminobenzoic acid (PABA) which can be incorporated into study foods then assessed through urinary excretion (Higgins and Mattes, 2019). Thresholds for determining an acceptable level of compliance (e.g., >95% of provided foods) can be established *a priori*.

### Limitations and Lessons Learned

Controlled feeding studies are challenging and resource intensive, and participant burden can be substantial. In addition, outcomes measures are typically limited to surrogates. However, the high degree of dietary precision is ideal for testing efficacy. There may be unanticipated challenges that arise, such as changes in food product formulations, electrical power outages, or equipment failure. Differences in habitual diet prior to participation can also impact responses to the controlled diet. As such, controlled diet lead-in periods may be warranted. Controlled diet lead-in periods can also be helpful in identifying participants mostly likely to be non-compliant to the intervention. Careful advance study planning and pilot-testing procedures before study implementation can help to insure efficient study operations, and enable rapid responses to the inevitable challenges that often arise in human research. Importantly, collaboration with experienced clinical nutrition investigators can be an important means for designing, implementing, and interpreting the findings from high quality controlled feeding studies.

## Contributions of Controlled Feeding Studies to Nutrition Science and Integrative Physiology

Controlled feeding studies provide a powerful means for testing proof-of-concept and efficacy studies to determine if one or more nutrients or a specific dietary pattern, given in a known amount, exert an important impact on physiology/metabolism. The rationale for such studies may be to determine the relevance of observations in animal models to humans the mechanism(s) responsible for observations made in observational or behavioral intervention studies. Importantly, the findings of controlled feeding studies may be considered together with epidemiological and behavioral intervention studies to inform DGA. We summarize below the findings of several examples of controlled feedings studies that have informed DGA and/or provided important insight into integrative physiology. Our intent is not to critique these individual studies but rather to use them as exemplars on how controlled feeding studies can be uniquely insightful.

### *Trans* Fatty Acids and Cardiovascular Disease Risk

Partially hydrogenated fats were increasingly used in the 1960s, 1970s, and 1980s as the food industry moved away from animal fats and tropical oils in response to public health recommendations (Mozaffarian et al., 2006). While the impact of dietary fatty acids, particularly saturated fatty acids, on CVD risk is unsettled, there is considerable evidence to support the DGA recommendations for reducing *trans* fatty acids in the diet to as little as possible (U. S. Department of Health and Human Services and U. S. Department of Agriculture, 2015). In 1990, Mensink and Katan (1990) reported on a seminal study in which 59 apparently healthy men and women were randomized to consume each of three diets comprised of 10% oleic acid (one *cis* double bond), saturated fatty acids, and *trans* fatty acids (*trans* isomer of oleic acid) for 3 weeks. The nutrient composition of the dietary conditions were otherwise closely matched. The major outcomes were serum lipoprotein concentrations. There was no washout period included because the authors prior observations suggested that serum lipoprotein concentrations stabilized within 2 weeks after a dietary change (Mensink and Katan, 1987). The major finding was that *trans* fatty acid intake increased low density lipoprotein cholesterol (LDL-C) and decreased HDL-C compared with the oleic acid diet. The increase in LDL-C was significantly less than observed with the saturated fat diet. In addition, both *trans* fatty acid and saturated fatty acid intake raised triglyceride concentrations. The adverse impact of *trans* fatty acid intake on lipid and lipoprotein concentrations was confirmed in a large meta-analysis of controlled feeding studies (Mensink et al., 2003) and subsequently in controlled feeding studies in a United States sample (Judd et al., 1994). Importantly, the *trans* fatty acid intake level used in this latter study was similar to that consumed by the United States population at the time (∼2–4% of total energy intake). Finally, a meta-analysis of prospective observational studies indicated that a 2% increase in energy intake from *trans* fatty acids was associated with a 23% increase in the incidence of coronary heart disease (Mozaffarian et al., 2006).

Tiburon, California started a national movement when in 2003, 18 restaurants voluntarily stopped using *trans* fats in their cooking oil (Staats, 2007). New York City Department of Health and Mental Hygiene followed suit and in 2006 approved the first legal restriction of *trans* fatty acids (Angell et al., 2009). The first national recommendations to limit *trans* fatty acid intake in the United States to as low as possible appeared in the 2005 DGA (U. S. Department of Health and Human Services and U. S. Department of Agriculture, 2015). In 2006, the Food and Drug Administration mandated that the *trans* fatty acid content in a serving of food be displayed on the Nutrition Facts panel of all packaged foods. This regulatory action appeared to motivate food manufacturers to reformulate many of their products to reduce the *trans* fatty acid levels, increased public awareness, and resulted in efforts by cities and states to place limits on *trans* fatty acid content of restaurant foods (Angell et al., 2009). Denmark has virtually eliminated *trans* fatty acids from their food supply, and the strict limits on *trans* fats were followed by a decline in CVD morality (Restrepo and Rieger, 2016). In 2015, the Food and Drug Administration determined that the Generally Recognized As Safe (GRAS) designation will no longer be applied to partially hydrogenated oils (FDA, 2015). More recently, the World Health Organization has released plans to altogether eliminate *trans* fats from the global food supply (World Health Organization, 2018).

### Ultra-Processed Foods and Energy Intake

The consumption of ultra-processed foods has increased dramatically and now comprise more than half of total energy and approximately 90% of the energy intake from added sugars in the United States diet (Martinez Steele et al., 2016). There is accumulating evidence that ultra-processed food consumption is associated with poor dietary quality, low satiety potential and a number of poor health outcomes (Fardet, 2016; Monteiro et al., 2018; Rico-Campa et al., 2019; Srour et al., 2019). However, until recently a causal role of consumption of ultra-processed foods in contributing to deleterious (or beneficial) effects on health could not be established. Recently, Hall et al. (2019a) conducted a randomized controlled feeding trial to test the effects of ultra-processed compared with unprocessed diets on *ad libitum* energy intake. To address this, 20 normal weight and overweight volunteers were confined to a metabolic research unit for 28 days and, in a randomized crossover fashion, were provided *ad libitum* access to diets comprised of ultra-processed or unprocessed foods for 2 weeks. The alternate diet was provided immediately following for another 2-week period. Participants were provided with meals three times each day, with a 60 min period time period given for consumption. Snacks consistent with each of the respective diets and water were provided throughout the day. The 7-day menu rotation was designed to be similar in macronutrients, sugar, fiber, sodium, and energy density but differed in the proportion of calories from ultra-processed and unprocessed foods. Ultra-processed foods were classified using NOVA (Monteiro et al., 2018). The major finding from this study was that a diet comprised of ultra-processed foods resulted in increased energy intake and weight gain compared with the diet comprised of unprocessed foods. The foods consumed in the ultra-processed food diet had high energy densities, but there were no differences in the pleasantness or familiarity ratings of the foods provided in the two diets. In addition, sodium intake was higher but fiber intake was similar in the ultra-processed compared with unprocessed food diet. There were no significant differences in the changes in glucose tolerance or insulin sensitivity with the interventions although it is possible that a study over a longer duration may have yielded a different outcome.

The Dietary Guidelines for Brazilians recommend that ultra-processed foods be avoided because their formulation and presentation results in overconsumption and, in turn, many unprocessed or minimally processed foods are displaced from the diet (Ministry of Health of Brazil, 2015). Importantly, the results of large prospective cohort studies have suggested that higher consumption of ultra-processed foods is associated with an increased risk of CVD (Srour et al., 2019) and mortality (Kim et al., 2019; Rico-Campa et al., 2019). Although the United States DGA do not specifically recommend avoiding of ultra-processed foods, these recent findings have important policy implications. However, we should emphasize that the classification of ultra-processed foods using NOVA (Monteiro et al., 2018), as utilized by Hall et al. (2019a) and recent cohort studies (Srour et al., 2019) and mortality (Kim et al., 2019; Rico-Campa et al., 2019) ais somewhat controversial (Gibney, 2019). In addition, research using controlled feeding designs that include lead-in and washout periods as well as and diets that are matched for types of sugar, fiber, and fat is needed to confirm the findings of Hall et al. (2019a). Further study is needed to determine if there is a dose-response relation between ultra-processed food consumption and energy intake, and to determine if the increased energy intake during consumption of a diet high in ultra-processed foods persists over longer periods of time. Finally, more research is also needed to identify causal mechanisms by which the consumption of ultra-processed foods increases energy intake, if confirmed.

### Dietary Approaches to Stop Hypertension: A Story of Successful Translation

The DGA recommend consuming a health dietary pattern and the DASH is provided as example of a healthy dietary pattern that emphasized consumption of fruits, vegetables, low-fat dairy foods, whole grains, poultry, fish, and nuts. The study was a multi-center (four sites) randomized controlled feeding trial to test the efficacy of a DASH dietary pattern on BP in patients with normal or stage 1 hypertension (Appel et al., 1997). The rationale behind the trial was that prior studies relied on the manipulation of individual nutrients, macro- or micro-nutrients, or food groups to lower BP have produced small and inconsistent effects. Along these lines, nutrients consumed together may produce additive physiologically and statistically significant effects. Many studies prior to DASH testing the effects of diet on BP involved behavioral interventions where adherence is problematic. The study protocol required that all subjects consume a control diet that was comprised of 55% carbohydrate, 30% fat, and 15% protein for 3 weeks. Three servings of low-fat dairy were added to the high fruit and vegetable diet to increase potassium and calcium and, thus, offset sodium effects. In addition, the level of energy intake was designed to allow subjects to maintain a stable weight throughout the course of the intervention. The latter was critical to the success of the study given the well-documented impact of weight loss on BP (Aucott et al., 2009). At the end of 3 weeks, subjects were randomized to one of three conditions, a control diet similar to the lead-in, a diet high in fruits and vegetables, and a combination or DASH diet for an additional 8 weeks. Sodium intake was the maintained at the same level in the three groups. A requisite for having a multi-center controlled feeding study was that all of the elements necessary for successful execution of a multi-center trial must also apply to the food service and menus. To address this issue, identical menus were provided at each of the four centers in manner that was palatable to a diverse population. The impact of food procurement, storage, preparation, and meal delivery was also minimized among the centers. Food groups, rather than supplements, were utilized to reach the target nutrient pattern. Foods were incorporated into the menus if they were rich in the selected nutrients and typically consumed by the population. The primary outcome variable was the change in BP with the intervention. The major finding from the DASH trial was that, among those with hypertension, the combination (i.e., DASH diet) reduced systolic and diastolic BP by 11.4 and 5.5 mmHg more, respectively, than the control diet. The reductions in those without hypertension were 3.5 and 2.1 mmHg, respectively.

One of the major advantages of the controlled feeding design used in the DASH trial was that it allowed for mechanisms to be explored. Akita et al. (2003) reported in an ancillary study that the reduction in BP with the DASH diet was due, at least in part, to a leftward shift in the renal pressure-natriuresis curve that describes the relation between BP and urinary sodium excretion. That latter suggests improved renal excretory function following consumption of the DASH diet.

The PREMIER study was designed to test the effectiveness of a lifestyle modification program including the DASH diet under real-world conditions, in adults with prehypertension as well as in those with stage 1 hypertension (Appel et al., 2003). The primary outcome, systolic BP, declined by 4.3 mmHg in the lifestyle modification program that included the DASH diet. Taken together, the DASH diet can be considered success story for translational research given the consistent evidence across multiple study types and its broad implementation and dissemination, and incorporation into the DGA.

### Macronutrient Composition of the Diet

High intake of dietary fat has a long history of being implicated as a cause for numerous chronic diseases (Grundy et al., 1982; Kritchevsky, 1998; Sacks et al., 2017; Fattore and Massa, 2018; Ludwig et al., 2018b). Beginning in 1957, the American Heart Association published reports at ∼3–5 year intervals summarizing and eventually providing dietary guidelines related to dietary fat intake for preventing and treatment heart disease and stroke. In 1977, a report by the United States Senate Select Committee on Nutrition and Humans Needs called on Americans to reduce calorie intake and the consumption of total and saturated fat and to increase the intake of carbohydrates. Subsequently, dietary recommendations prioritized reducing dietary fat intake (Jahns et al., 2018). The Food Guide Pyramid, released by the United States Department of Agriculture in 1992, placed 6–11 servings of bread, cereal, rice and pasta at the base and sparing use of all fats and oils at the top. In turn, the food industry was called upon in the United States Healthy People 2000 report to market products that were reduced in total and saturated fat (U.S. Department of Health and Human Services, 2019).

Ludwig and Ebbeling (2018) has put forth the “carbohydrate-insulin model” in attempt to offer a physiological explanation for why obesity rates have increased since the 1970s. According to this idea, “recent increases in the consumption of processed, high-glycemic-load carbohydrates produce hormonal changes that promote calorie deposition in adipose tissue, exacerbate hunger, and lower energy expenditure.” “This physiological state is hypothesized to increase hunger and food cravings, lower energy expenditure, and predispose to weight gain, especially among those with inherently high insulin secretion” (Ebbeling et al., 2018a). To address this, Ebbeling et al. (2018a) conducted an ambitious controlled feeding study over a 20-week duration to compare the effects of diets with different proportions of diet fat and carbohydrate on total daily energy expenditure during weight loss maintenance. Following a 10-week lead-in period to restrict energy intake (mixed macronutrient) to reduce body weight by 10%, overweight and obese adults were randomized to low (20% of total energy), moderate (40%) and high (60%) carbohydrate diets designed to maintain the achieved weight loss for an additional 10 weeks. All food was provided in free-living participants via a unique collaboration between the investigators, Framingham State University, and a food service contractor. Participants consumed at least one meal/day while supervised in an on campus facility. The remainder of the meals were packed with snacks and sent home with the participants. The details have been published previously (Ebbeling et al., 2018b). The change in total daily energy expenditure via doubly labeled water from the end of the 10-week lead-in period to the end of the 10-week weight maintenance period was the primary outcome. The change in total daily energy expenditure was 91 kcals/d and 209 kcals/d greater in the moderate and low, respectively, compared with high carbohydrate diet. Interestingly, the effect of diet composition on total daily energy expenditure was greatest in those with the highest insulin secretion at baseline (i.e., prior to lead-in weight loss period). Taken together, these data provide important new insight on the potential role of low carbohydrate, low fat diets in obesity treatment. However, it is important to emphasize that differences of opinion exist on the analysis and interpretation of the study (Hall et al., 2019b).

### Dietary Sodium and Blood Pressure

Current national guidelines recommend Americans reduce dietary sodium to less than 2300 mg/d. However, there is disagreement in the scientific community and in 2013 the Institute of Medicine was commission by the Centers for Disease Control and Prevention to evaluate the evidence linking dietary sodium to health outcomes, particularly hard rather surrogate endpoints (Institute of Medicine, 2013). The Committee was specifically charged with considering the potential benefits and harms of reducing daily sodium intake to between 1500 and 2300 mg/d with a particular focus on high risk groups. In the general population, no conclusion could be reached regarding whether cardiovascular benefit or harm resulted from consumption of less than 2300 mg/d of sodium intake. There was no clear benefit or harm of sodium restriction to less than 2300 mg/d in the general population and potential harm in some clinical populations including congestive heart failure and diabetes. In general, the committee found reported studies in this area to be highly variable and considered much of it flawed. While not alone in its recommendation (Jones et al., 2018), the IOM committee recommended that “clinical trials might focus on examining the effects of a range of sodium levels on risk of cardiovascular events, stroke, and mortality among patients in controlled environments” (Institute of Medicine, 2013). Because adherence to sodium intake less than 2300 mg/d for greater than 6 months is challenging in behavioral intervention trials, the IOM Committee recommended a trial of sodium reduction be performed using a controlled environment where the content of sodium in prepared food could be manipulated.

In May 2017, a group of scientists and clinicians involved in hypertension research and treatment, respectively, met to consider the issue of sodium and CVD and a summary statement was published (Jones et al., 2018). The groups reached a consensus that a randomized controlled feeding trial evaluating the impact of dietary sodium on hard endpoints was the only likely means to resolve differences of opinion on existing evidence. Members of the active military and nursing home residents were considered as populations residing in setting where the sodium content of foods could be controlled. There were concerns raised with each of these possibilities that precluded a consensus being reached. There was, however, agreement that the federal prison population might provide the best setting to conduct such a trial. The strengths of this option included the ability to control dietary intake in a large population at multiple locations, the possibility of a randomized cluster design, age and racial/ethnic diversity of the population, and the ability to conduct the trial in an existing research infrastructure. Although there is an extensive literature on ethics of prison research and there was expected health benefit to the prisoners, there remained concerns among the group regarding ethical issues surrounding prison research and a question regarding whether an adequate number of sites could be enrolled. Such as study would seem unlikely to come to fruition given protections provided to prisoners as a vulnerable population under the Common Rule (45 CFR 46). The reader is referred to an updated review of the evidence and the establishment of a new category of Dietary Reference Intakes, referred to as the Chronic Risk Reduction Intakes (National Academies of Sciences Engineering and Medicine, 2019). The later was implemented as a result of recommendations from the Guiding Principles Report (National Academies of Sciences Engineering and Medicine, 2017).

### Validation of the Doubly Labeled Water Method

Controlled feeding studies have played a vital role in the validation of dietary and physical activity methods. The doubly labeled water method, considered the “gold standard” for measuring energy intake, as well as physical activity, in free-living individuals is one such example. The reader is referred Westerterp et al. (Westerterp, 2017) for background on theory and use of the doubly labeled water method. In 1982, Schoeller and van Santen (1982) compared the doubly labeled water method for estimating energy requirements with that derived from the intake-balance method, i.e., deriving energy requirements from controlled feeding to weight stability. To accomplish this, prescribed doses of ^18^O and ^2^H as water were provided to four participants. Urine samples were collected at baseline (for enrichment) and 14 days after dosing for analysis of the differential elimination rates of the isotopes which, in turn, provides a measure of carbon dioxide production. When used with the average respiratory quotient (approximated from the food quotient) the ratio of carbon dioxide production and oxygen consumption provides for the calculation of energy expenditure. During the interim period, the participants were provided a weight maintenance diet to derive energy and water intake under free-living conditions. Energy requirements during this period were calculated from energy balance via the sum of energy intake and the change in body stores (estimated from body composition changes). Doubly labeled water-determined energy expenditure differed by 2% (C.V. = 6%) from that derived from the intake-balance method. Subsequent validation against whole-room calorimetry supported the accuracy the doubly labeled water method with a precision of 2–8% (Schoeller, 1988) across a range of activity levels (Westerterp et al., 1988). The technique has been broadly used in obesity research, validation of dietary intake and physical activity assessments, measurement of energy requirements in diverse populations, as well as in the study of human growth.

## Important Role of Controlled Feeding Studies in the Future

### Rigor and Reproducibility

Nutrition science has been subject to considerable criticism and controversy. Rigor involves the scientific practices that enhance the likelihood that an observation will be reproducible (Casadevall and Fang, 2016; National Institutes of Health, 2019). Reproducibility, i.e., the ability to reproduce a scientific finding, is dependent on the rigorous application of the scientific method to minimize bias in the experimental design, methodology, analysis, interpretation and reporting (Collins and Tabak, 2014; National Institutes of Health, 2019). Controlled feeding studies offer an opportunity to test well-founded hypotheses under highly controlled conditions. The more rigorous the elements addressed above are implemented and executed, the more reproducibility should be enhanced. Blinding can be difficult in controlled feeding studies. In addition, appropriate oversight and standard (Moher et al., 2001) and sufficiently detailed reporting of the methodology in publications describing research involving controlled feeding studies (Mitchell et al., 2015) is essential to maximizing reproducibility. Importantly, registration of controlled feeding studies (e.g., clinicaltrials.gov) is necessary for transparency. Of course, reproducibility of a rigorous experimental design can become compromised for other reasons such as introduction of biases^2^ prior to and following randomization.

### Dietary Control During Lead-in Periods

Dietary control during lead-in periods can be a valuable design feature of in the conduct of controlled feeding studies. In our experience, dietary control during lead-in periods can serve to reduce variability of outcome variables prior to intervention. Even with exclusion of participants on the dietary extremes, considerable variability in nutrient consumption often remains in participants enrolled in controlled feeding studies. Controlling dietary intake during a lead-in period can serve to bring participants toward a common baseline. Dietary control during lead-in periods can also serve as a means to screen non-compliant participants, e.g., those either unwilling to consume all of the food provided or those who fail to disclose aversions or allergies to key foods/nutrients during screening.

### Dietary Patterns and Overall Diet Quality

There has been increasing attention being given to emphasize understanding the impact of dietary patterns and/or overall diet quality on chronic disease risk, rather than a focus on individual nutrients or foods. In addition, there is growing recognition that changes in dietary habits are often characterized by substitution effects; high consumption of some foods is often associated with lower consumption of others. Dietary intake can be manipulated to conform to a particular dietary pattern using controlled feeding studies. The latter may be defined by a healthy eating index (HEI) (Kennedy et al., 1995) or alternative HEI (McCullough et al., 2002), metrics that quantify the degree to which a diet conforms to the DGA (scored 1–100). The reader is referred to Krishnan et al. (2018) who recently tested the impact of a dietary pattern that conformed to the 2010 DGA in individuals at risk for cardiometabolic disease. The experimental and control diets differed dramatically in HEI scores (98 vs. 62 out of 100). Similar metrics are available for studying the degree to which a given diet conforms with the DASH diet (Lin et al., 1999; Harmon et al., 2015) and the Mediterranean Diet (Trichopoulou et al., 1995; Harmon et al., 2015).

### Dietary Intake and Composition of the Gut Microbiome

Dietary patterns, specific foods, and constituents of foods have a distinct influence on the gut microbiota (Graf et al., 2015). Western diets, which are low in fiber and high in both fat and refined carbohydrates, have been associated with a permanent loss of specific bacterial taxa (Zinocker and Lindseth, 2018), as well as reduction in community diversity (Le Chatelier et al., 2013; Sonnenburg et al., 2016), and “dysbiosis” of the gut microbiota (Zinocker and Lindseth, 2018). Importantly, the composition of the microbiota can change rapidly (i.e., within 24 h) in response to short-term change in macronutrition content of the diet (Wu et al., 2011; David et al., 2014). In turn, long-term dietary changes, particularly high in protein and animal fat intake, can lead to alterations in the structure and activity of gut microbial communities (Wu et al., 2011).

There are a number of challenges with characterizing the effects of diet on the human microbiome. Such challenges were recently highlighted in a summary of a National Institutes of Health workshop focused on improving rigor and reproducibility in research focused on the colonic microbiome (Klurfeld et al., 2018). These include poor adherence to diets, relative inaccurate characterization of dietary intake and composition, the reciprocal nature of dietary changes when isocaloric conditions are maintained, and high costs. In general, observations studies are limited to associations. As such, it is recommended that controlled feeding studies are needed along with model systems as their combination may be more insightful than either alone.

The effort to understand the role of the gut microbiota in contributing to physiological and metabolic phenotypes can be complicated by habitual dietary intake. For example, much of the available evidence suggesting that exercise modifies gut microbial composition is based on observational and cross-sectional studies where differences across strata of physical activity levels or between exercise trained and sedentary individuals is confounded by variability in habitual dietary intake (Mitchell et al., 2019). Future studies on the relation between physical activity or other interventions/phenotypes and the composition and function of the gut microbiota and physiological phenotypes should carefully consider the need to control habitual dietary intake.

## Conclusion

Nutrition science has made vital contributions to improving human health primarily by identification of nutrient requirements and elimination of deficiencies. What we eat has become as polarizing a topic as religion and politics in part because the contributions to preventing and treating chronic disease have been more limited. There are unlikely to be randomized controlled trials which test the effect of diet on hard outcomes as many would desire; the best available evidence will need to be considered. Unfortunately, there is a lack of validated biomarkers in the causal pathway for most chronic diseases. Controlled feeding studies will be important in this regard and together with epidemiological studies and behavioral interventions can be used to develop evidenced-based recommendations. Preclinical studies may also be used to elucidate the biological mechanisms and pathways mediating the effects of nutrients. Importantly, individual studies are neither necessary nor sufficient for overall actionable evidence (Mozaffarian and Forouhi, 2018). Evidence from a variety of study types should be used to provide complementary evidence-based nutrition guidance (Navia et al., 2010; Magni et al., 2017; Mozaffarian and Forouhi, 2018). Importantly, controlled feeding studies can also be used to improve the rigor and reproducibility of studies of human integrative physiology and metabolism. Additional resources for conducting controlled feeding studies are available (see Table 1).

**TABLE 1:** Additional resources: designing and implementing controlled feeding studies.

**Source**	**Link and relevant contents**
Dennis et al., 1999	https://books.google.com/books/about/Well_controlled_Diet_Studies_in_Humans.html?id=5lYTAQAAMAAJ Comprehensive resource book covering all aspects of controlled feeding studies. Includes chapters which address facilities and equipment, estimating facility space needs, studies in children, dietary checklists and forms, and staff position descriptions
Van Horn and Beto, 2019	https://www.eatrightstore.org/product-type/books/research-fourth-edition Nutrition research methodology resource book. Chapter 8 (pp. 126–152) addresses guidelines for developing and implementing clinical nutrition studies. Other chapters address dietary assessment, food composition databases, appetite and biomarker assessment research, and dietary supplementation research
Most et al., 2003	https://www.ncbi.nlm.nih.gov/pubmed/12778045 Article which compares the design and management of a controlled feeding vs. behavioral intervention study (i.e., DASH vs. PREMIER). Describes the knowledge and skills required for research dietitians and kitchen staff involved with planning and implementing controlled feeding studies
Hall and Most, 2005	https://www.ncbi.nlm.nih.gov/pubmed/16182647 Research article which describes an investigation of the effects of study design factors (e.g., participant’s demographic factors, diet duration, days in menu cycle, allowance of alcohol) on participant’s adherence to controlled diets. Types of diet deviations (e.g., eating all study foods vs. eating foods not on the controlled diet) were also assessed
Sacks et al., 1995	https://www.ncbi.nlm.nih.gov/pubmed/7795829 Article which describes the rationale for DASH trial and study diets; compares the composition of the three controlled experimental diets (e.g., control, fruits/vegetables, DASH). Describes the controlled diet run-in period, which was used to screen for dietary adherence. Procedures for assessing self-reported and objective measures of dietary adherence are presented
Swain et al., 1999	https://www.ncbi.nlm.nih.gov/pubmed/10450295 Article presents the steps taken to develop the DASH trial’s experimental diets, multi-center aspects of the trial (e.g., methods to standardize diet procedures across sites), palatability evaluation, and procedures for validating the experimental diets using food composition analyses
National Cancer Institute	https://dietassessmentprimer.cancer.gov/learn/observation.html Online resource; other sections of this site address dietary assessment methods, food composition databases, software for dietary analysis

## Author Contributions

KD and BD conceived the idea for the manuscript, agreed on content, contributed to the writing and editing the manuscript, and approved the final draft of the manuscript.

## Conflict of Interest

The authors declare that the research was conducted in the absence of any commercial or financial relationships that could be construed as a potential conflict of interest.

## Footnotes

1 https://www.servsafe.com/ServSafe-Food-Handler

2 http://catologofbias.com
